# Cauda Equina Syndrome: A Review of 15 Patients Who Underwent Percutaneous Transforaminal Endoscopic Lumbar Discectomy (PTELD) Under Local Anaesthesia

**DOI:** 10.5704/MOJ.2007.019

**Published:** 2020-07

**Authors:** A Krishnan, R Kohli, D Degulmadi, S Mayi, R Ranjan, B Dave

**Affiliations:** Department of Spine Surgery, Stavya Spine Hospital and Research Institute, Ahmedabad, India

**Keywords:** cauda equina syndrome, transforaminal endoscopy, percutaneous, minimal invasive spine surgery

## Abstract

**Introduction:**

To analyse the results of Cauda Equina Syndrome (CES) operated by Percutaneous Transforaminal Endoscopic Lumbar Discectomy (PTELD).

**Material and Methods:**

The study is a retrospective series of 15 patients operated by PTELD. Bladder dysfunction was classified as incomplete CES (CESI) and complete CES retention (CESR). Bladder / motor recovery rate and its timing, Oswestry Disability Index (ODI), Visual Analogue Score (VAS), patient satisfaction index, and sexual dysfunction were used to measure the outcome objectively. Additionally, in CESR patients, post-void residual (PVR) urine was measured by sonography. Complications and technical problems were noted.

**Results:**

There were ten patients of CESI and five patients of CESR. The average follow-up was 20.33(12.05) months. Bladder symptoms recovery was 100%, and motor recovery was 80%. VAS for back pain recovered to 0.53(0.52) from 8(2.39). VAS for leg pain recovered to 0.13(0.35) from 9.20(1.32). ODI improved to 6.07(2.85) from 77.52(13.20). The time to the recovery of bladder function was 1.47(1.55) days. All CESR patient’s abnormal PVR urine was normalised at five weeks post-operative. No complications were reported. However, five technical executional problems occurred.

**Conclusion:**

PTELD can be considered for CES treatment due to its substantial and quick recovery advantages. However, more evidence support is needed to make it a practice recommendation.

## Introduction

Cauda equina syndrome (CES) is one of the most serious and complicated spinal pathologies^1, 2^. It has been reported as the extreme presentation of 1-3% of Lumbar Disc Herniation (LDH) patients^2-4^. Conventionally, treatment for CES is open laminectomy/discectomy (MED: Microscopic Decompression)^5^. Although minimally invasive approaches have been reported, they are often not recommended due to perceived manipulations required in a smaller approach. Additionally, there are high chances of residual disabilities of limb and/or bladder and/or sexual dysfunction leading to unhappiness and medico-legal litigations^3, 6^.

The use of Percutaneous Transforaminal Endoscopic Lumbar Discectomy (PTELD) for treating CES is sparsely mentioned in the literature^7-13^. Indications of PTELD have evolved significantly and in India, the catalyst of the evolution was the mentorship of Dr Gore^11, 14^. The advantages of PTELD are the use of local anaesthesia, day-care, least pain, speedy recovery, preservation of muscles, stability, low blood loss, and minimal post-operative complications^8-10, 13, 14^.

There are other added advantages of PTELD in CES that are worth considering. There is less manipulation of compromised neural tissues, and urgent surgery is possible without lengthy pre-anaesthetic preparations^8, 9, 13, 14, 15, 16^. There are also the significant considerations of pain reduction and enhanced recovery with minimally invasive surgeries^17^. Recently, few articles on PTELD in CES have been published that quote unbelievably excellent outcomes, the majority of which reveal surprisingly quick and complete recoveries^8-12^. We report our retrospective non-consecutive case series and experience of PTELD in CES due to LDH.

## Materials and Methods

This study was a retrospective analysis of all operated PTELD cases. Informed consent was taken in all cases, which included the option of open surgery if optimal decompression fails. The study was conducted from January 2014 to December 2018. Inclusion criteria were a single level LDH with one or more of the following red flags: (1) bladder and/or bowel dysfunction, (2) reduced sensation in the saddle area, (3) sexual dysfunction with a possible neurologic deficit in the lower limb (motor/sensory loss, reflex change)^1, 4^.

Hospital medical records and image databases were analysed. Patient demographics reviewed included age, gender, back and/or leg complaint (acute, sub-acute or chronic), onset/duration of bladder problems, and neurological status. MRC (Medical Research Council) grading scale of 0 to 5 was used in assessing motor power. A significant weakness score was assigned if the muscle power was less than grade three.

CES classification: CES was categorised into two types. The first was CESI (incomplete), in which patients had a spectrum of urinary difficulties such as hesitancy, straining, low-pressure stream, and/or increased frequency. The second was CESR (with retention)^6^. Ultrasonographic measurement of post-void residual (PVR) urine was done in all CESR patients with measurement of more than 200ml considered positive^18^. Additionally, the bladder dysfunction of CES presentation was classified as either within 24 hours or more than 24 hours.

LDH Presentation acuteness: Patient presentation of LDH was classified as acute (within 48 hours), sub-acute (within one month), or chronic. Chronicity was labelled when the patient came in with a progressive lower back pain (LBP) or radiculopathy of more than three months duration. The onset of bladder dysfunction signaled the start of CES.

MRI (Magnetic Resonance Imaging) scans were assessed for the presence of a complete myelo-block. Overt instability was ruled out and the radiological type of intra-canalicular LDH was labelled according to the axial view (central or para-central) and sagittal view (for migration). Lateral recess stenosis (LCS), if present, was noted. Calcifications (CC) of disc/annulus or end plate spurs were noted and confirmed in intra-operative findings.

Surgical techniques: An independent anaesthesiologist was present during the whole surgical procedure. The surgery was done with local anaesthesia (LA) and conscious sedation. The patient was put on protected prone position and received supplemental oxygen. Forty-five minutes before surgery, intramuscular midazolam (0.05mg/kg) and diclofenac were given. Half a dose of titrated infusion of dexmedetomidine (0.5-1 mcg/kg) was given slowly with an intravenous dose of 1mcg/kg of fentanyl bolus ten minutes before the surgery. This was followed by additional doses as needed.

A uni-portal approach was used. An imaginary line was drawn to the annular puncture site and the skin site was marked for the surgical trajectory planned. The angle was between 0° and 30°, and the puncture point was 12-16 cm from midline depending on the basic technique used. The intended needle entry tract was infiltrated with 1% lidocaine plus bupivacaine at 1:1 ratio. A 16-gauge needle was inserted fluoroscopically in the Kambin’s triangle with continuous patient’s feedback and 1.5ml further infiltration on the annulus. A 7mm incision was made and followed with tapered dilating trocar. The beveled working cannula was railroaded, and then through that the endoscope was introduced. The removal of the offending compression was done with any of the inventory as needed [Carl Storz-Gore System-Germany / Maxmore system-Germany or Richard wolf system-Germany]. Prolapsed disc excision was done along with compressive ventral tissues. The techniques employed depended upon the pathology and were standard basic techniques of outside in (OI) or inside out (IO) or FEE (flat epidural entry), with modifications as needed.

Inside Out (IO): An approach at a 15 to 20° angle was taken. The disc was pierced, and the sub annular disc was removed before cutting the annular anchorage and working in epidural space^13, 14^.

Outside In (OI): Blind non-visualised reamed foraminoplasty using Maxmore Tom Shidi reamers was done to reach epidural space directly^15^.

FEE (flat epidural entry): The flat approach was taken nearly at an angle of 0° landing in the epidural space avoiding the piercing of the disc and facet reaming^16^.

An endoscopic visualised BF (burred foraminoplasty) or OI approach was taken when there was a technical requirement to reach more dorsal. Nouvag system [Goldache, Switzerland] was used. When CC was contributing to the ventral stenosis endoscopic osteotomes and burr were used (CVD: Calcified Ventral Decompression). In bilaterally symptomatic cases, if the adequacy of decompression was not satisfactory from index uni-portal approach (UPA) due to limited reach to opposite ventral side, then in the same stage, opposite side transforaminal approach was taken (Bi-lateral, Bi-portal approach; BLBP). The decompression endpoints were by complete visualisation of the roots, dural sac, probing, dural pulsations, irrigation flutter, and cough impulse.

Sub-cuticular stitches were taken, and all patients were sent for MRI. All patients were mobilised as per their tolerance and limb power. Requisite patients were advised to undertake passive/active physiotherapy. The urinary catheter was not done pre-operatively for any patient except the ones needing GA. The level of surgery, operating time (OR Time), and estimated blood loss (EBL) were noted. All patients were reviewed based on the time between the onset of CES to surgery, the length of hospital stay (LHS), and the months of follow-up. The evaluation of neurological and functional outcomes was done using validated measures.

ODI: In ODI, nine components (out of ten) were used, and sexual dysfunction was not used. Total added score as a percentage is expressed. Ten-point Visual Analog Scale score (VAS) scale for LBP and leg pain was used.

Motor and Bladder Improvement: Improvement beyond MRC grade three was considered as recovery. The bladder outcome (recovered or not) was assessed. Bladder status was noted as recovered to normal, recovered to CESI status, or remaining as CESR with the time (days) to the recovery.

Sexual dysfunction scoring: Sexual dysfunction scoring on the basis of the SHIM (Sexual Health Inventory in Males) and a non-validated questionnaire to assess female sexual dysfunction was collected as a follow-up only. The SHIM is a five-item questionnaire validated as a screening tool for erectile dysfunction^6, 19^. However, in conservative societies like India, a woman is expected to maintain silence when confronted with issues of her own sexuality. So, instead of available validated female sexual dysfunction scores, we used a non-validated self-assessment questionnaire. The females were asked to give an overall score to categorise their sexual function as good, fair, or poor considering factors of frequency, satisfaction, dryness, and pain^6^.

Overall satisfaction: Patient Satisfaction Index was used as a self-assessment tool to determine the overall satisfaction outcome with 3 parameters - one highly satisfied, two moderately satisfied, and three not satisfied^6^. The quality of residual LBP was categorised into NILBP (non-instability LBP) and ILBP (instability-related LBP). If LBP was present, as a constant backache not related to workload and not aggravated by loading, which felt more like a pulling or stiffness in the back, it was labeled as NILBP. The pain in ILBP was typically associated with positional change, such as standing up from sitting, bending forward, and floor activities, or related to workload aggravation^6^. Complications and technical problems were noted. The following recovery rates were assessed:

Bladder recovery rate = [Number of patients with complete bladder recovery/Number of patients with pre-operative bladder dysfunction] * 100 %

Motor recovery rate = [Number of patients with motor recovery (MRC>grade 3)/Number of patients with preoperative motor weakness (MRC <grade 3)] * 100 %

Statistics: Patient demographics and characteristic categorical variables were analysed, and the mean (standard deviation) for all applicable variables were calculated. Each category was compared by using appropriate statistical tools such as the Pearson correlations, unpaired Student t-test, and paired t-tests. Statistical analysis was performed with IBM SPSS software ver. 20.0 [IBM Corp, Armonk, NY, USA]. A p-value of <0.05 was considered to be statistically significant.

## Results

A total of 15 non-consecutive patients (13 males and 2 females) were operated for CES by the author (AK). None needed a conversion to open surgery intra-operatively or later. Their demographic presentation variables were summarised (Table I).

**Table I:** Demographic and Pre-operative variables

S No	Age	Sex		Level	Duration (Days)	Speed of onset Back/leg symptoms	Bladder Affection (hrs)	Numbness	LBP VAS	LL VAS	Motor weakness	Side Rt /Lt)	ODI	Classification CESI(i)/ CESR(r)	SA
1	57	M	PC	L45	35	SA	10	+	7	9	EHL EDL Rt	Rt	71.1	I	_
2	63	M	C	L45	50	SA	72	+	8	8	NA	Lt	63.2	I	_
3	21	M	C	L45	150	C	72	+	9	10	EHL EDL BL	BL	71.1	I	_
4	45	M	M PC	L23	30	SA	48	+	9	10	TA Rt	Rt	63.2	I	+
5	33	M	C	L45	90	C	72	+	10	9	NA	Lt	95.6	I	_
6	30	M	C & C	L45	60	SA	48	+	9	10	NA	Rt	84.4	I	_
7	45	M	M C	L45	120	C	72	+	8	9	EHL EDL BL, TS Rt	Rt	86.7	R	+
8	44	M	C	L45	180	C	48	+	8	9	TA Rt	BL	66.7	R	+
9	45	M	C	L45	86	SA	144	+	10	10	TA Lt	Lt	71.1	I	_
10	17	M	C	L23	2	A	36	+	0	5	Toes,Foot, hip, knee BL	BL	95.6	R	+
11	27	F	M C	L5S1	60	SA	8	+	7	9	TS Rt	Rt	66.7	R	+
12	23	M	C & C	L34	150	C	30	+	8	10	NA	BL	63.2	I	+
13	39	F	C	L5S1	70	SA	60	+	9	10	TS BL	BL	91.1	I	_
14	22	M	C	L45	48	A	24	+	9	10	NA	Rt	93.3	I	_
15	67	M	C & C	L45	10	A	6	+	9	10	EHL EDL Lt	BL	95.6	I	+

Type of Disc prolapse (Central C / Paracentral PC / Migrated M / Calcified c), Acute (A), Chronic (C), Subacute (SA), VAS Visual Analog Score, ODI-Oswestry Disability Index, Rt (Right) / Lt (left) / BL (Bilateral), CES Bladder affection Classification: CESI (lmpending-1) / CESR (Retention-R), TA-TibiaMs Anterior TS-Tendoachillis, EHL EDL-Extensor hallucis/ Extensor Digitorum longus, Saddle anaesthesia / Paraesthesia (SA), NA-Not applicable

The age of patients was 38.5 (15.6) years. Acute onset was present in three patients. The symptom duration was 76.1 (53.2) days. The duration of onset of bladder dysfunction was 50 (35.3) hours. And, four out of fifteen patients presented themselves within 24 hours. LBP was present in 14; tingling numbness was present in all 15, bilateral affection in ten, motor weakness in ten, saddle affection in seven and CESR in five patients, respectively. PVR urine 264ml (43.9) was present in all five CESR patients.

All patients had LDH. The most common level of lesion in our study was L4-5 (n=10). Additional LCS was present in one patient. All the patients had complete myelographic block. None of the patients had any instability. Twelve patients had central LDH. Three had CC, which was annulus calcification in one and endplate spurs in two. Seven patients had additional co-morbidities of obesity, hypertension, and/or diabetes mellitus.

All patients, except for one who demanded general anaesthesia (GA), were operated under LA. All the patients received surgery within 24 hours of presentation. However, only four patients presented within 24 hours of the onset of bladder dysfunction. No midnight emergency surgery was done. Surgery was done using the basic techniques of IO (n=11), OI (n=02), and FEE (n=02), respectively. Both OI was needed in l5 S1 LDH. In three patients with CC, the “inside-out” technique needed additional technique modifications (CVD) for removal of the hardened tissue. Additionally, BF was needed in four (IO=3, OI=1) patients for improving the reach. Two patients needed BLBP for complete decompression. Post-operatively in all patients, MRI confirmed the decompression. The average EBL was 31.3 (9.2) ml and duration of surgery 84.3 (22.3) minutes, respectively. All patients were immediately mobilised.

The average follow-up period was of 20.3 (12.1) months. The pre-operative back pain VAS was 8 (2.4) and it reduced to 0.5 (0.5) during the final follow-up. The pre-operative leg pain VAS was 9.2 (1.3), and it nearly vanished post-operatively and was reduced to 0.13 (0.4) at the time of the final follow-up. The results across these variables were statistically significant (Table II).

**Table II:** Table showing peri-operative and Core Outcome variables

S No	Surgery minutes	IO, OI, FEE Standard (S), Additional BLBP, CVD, BF.		Bladder Recovery days	Motor Recovery	ODI POP	LBP VAS	LVAS	Patient satisfaction Index	LHS	Duties Resumption	Fup (Months)	Associated Comorbidity/
1	47	FEE	S	1	R	6.7	1	1	1	1	2	43	Nil
2	98	10	S	1	NA	4.4	1	0	1	1	2	17	HTN
3	105	10	S	1	R	8.9	0	0	1	1	2	18	Nil
4	101	10	S	1	R	2.2	1	0	1	1	2	23	Nil
5	55	FEE	S	1	NA	11.1	1	0	1	1	2	28	Nil
6	93	10	CVD	1	NA	11.1	0	0	1	1	2	14	Nil
7	91	10	BF	7	RW EDL	4.4	0	0	1	3	2	19	LCS.Catheter Needed, DM, HTN
8	114	10	BLBP	1	R	6.7	0	0	1	2	1	16	HTN, Obesity
9	108	10	BF	1	R	2.2	0	0	1	1	2	51	Nil
10	80	10	S	2	R	6.7	0	0	1	3	3	14	Obesity
11	42	Ol	BF	1	RW EHL & EDL	6.7	1	1	1	2	3	18	Obesity
12	79	10	CVD/BLBP	1	NA	4.4	1	0	1	1	1	15	Nil
13	68	Ol	S	1	N	2.2	1	0	1	1	2	12	Obesity
14	96	10	BF	1	NA	6.7	1	0	1	1	1	11	Recurrence leg back (Mild)
15	88	10	CVD	1	R	6.7	0	0	1	2	2	6	HTN, DM

Basic Standard Technique(S)-IO : Inside Out / Ol : Outside In / FEE: Flat Entry Epidural, CVD: Calcified ventral decompression / BLBP: Biportal / BF:Burred Foraminoplasty, R: Recovered / RW: Recovered with residual weakness, POP: Post-operative, LHS Length of Hospital Stay, Fup: Follow up, Oswestry Disability Index (ODI), Visual analogue score (VAS), HTN: Hypertension, NA : Not Applicable Transforaminal Endoscopy in Cauda-Equina Syndrome

All CESI patients had complete bladder recovery immediately (100%). Of the CESR patients, three did not have symptoms of bladder dysfunction at discharge. Two patients had some straining difficulty, which lasted for a week in one patient and 48 hours in the other. First of these patients had to undergo indwelling catheterisation post-operatively that was removed after 72 hours. This was the same patient who was operated under GA. PVR urine on the follow-up of all CESR patients at five weeks was nil. The time to the recovery of bladder function was 1.5 (1.6) days.

The bladder recovery rate was 100%, and the motor recovery rate was 80%. Significant muscle power recovered in the remaining two patients to grade four MRC (one tibialis anterior with triceps surae and other triceps surae only). But these two had weakness in the toes present, though improved from previous ‘0’ MRC grade to grade 2. Out of the ten patients with a substantial deficit, eight recovered. There was non-bothering tingling numbness in legs present in five patients at the final follow-up. Residual saddle affection remained in two patients, and five completely recovered.

**Fig. 1:**
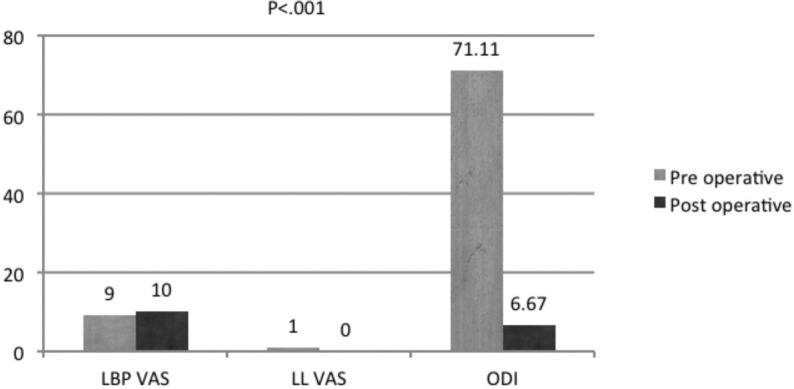
Bar diagram showing outcome parameters, Low back pain VAS, Limb VAS and ODI score.

The pre-operative ODI was 77.5 (13.2), and it improved quickly and progressively to 6.1 (2.9) at the final follow-up (Fig. 1). The patient satisfaction index was one in all patients. No NILBP was noted in any patient. ILBP was present in nine patients, which were occasional and were noted less frequently in those who followed lifestyle modification advice.

At follow-up, according to the SHIM scale, three patients had moderate erectile dysfunction. All of these three patients told that it was pre-existing. Female sexual function was good in both the patients.

**Fig. 2:**
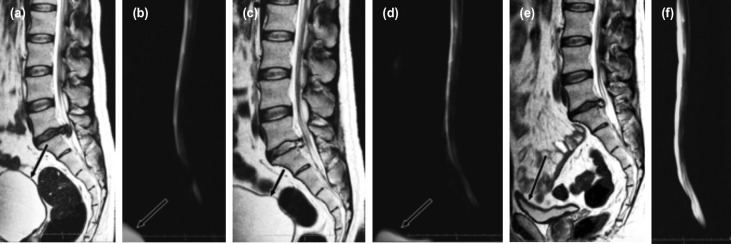
(a, b) MRI imaging in a patient with central big disc, complete myeloblock and CESR (Cauda Equina Syndrome Retention). Arrow showing distended bladder in MRI and myelogram. (c, d) Immediate post-operatively, patient had slow urine stream and needed straining to empty. The MRI showing a complete decompression and a clear myelogram. Arrow showing distended bladder post-operatively in MRI and myelogram as well. (e,f) MRI and myelogram on follow-up ten days shows complete empty bladder (arrow) without any clinical bladder dysfunction.

There were no major complications except technical problems of retrieving and decompressing in two patients who needed BLBP. One patient had more bleeding than usual. Two patients had fogging issues of the endoscope and needed to change to a second endoscope. One patient, after improvement of CES completely, at four weeks developed an episode of back pain (VAS 5). His activities were not affected, and a repeat MRI showed a recurrent small sequestrated disc extrusion. He became non-symptomatic again in three weeks through conservative means. One patient needed re-catheterisation and developed a urinary tract infection, which was conservatively treated, and eventually improved. One patient of CESR came back after 10 days with a mild LBP increase and was immediately subjected to MRI because of our anxiety despite no physical deterioration (Fig. 2).

## Discussion

CES was first reported in 1934 by Mixter and Barr, who pioneered it via a transdural approach^20^. Individuals in their fourth and fifth decades of life are prone to LDH and progression to CES^2, 21^. Our sample set for the study was mostly in their thirties, and men were more susceptible to be affected (n=13, 86.7%) than women. Various classification systems of CES have been reported without uniformity but bladder dysfunction is considered universal with rare sparing aswell^4, 22, 23, 24^. All our patients (n=15,100%) depicted classic symptoms of CES^4^.

MRI is the investigation of choice in CES^25^. All our patients (100%) had complete myelo-block, which in itself suggests severe compression. Small window surgeries are not recommended in CES and wider conventional opening may precipitate an instability^6, 26, 27^. Manipulation is dreaded and one report of spinal surgery itself as the cause of approximately 15% of the total numbers of CES brings to notice the true under-reporting in literature^28^. Trans-dural technique to avoid retraction has also been reported^1, 20^. One case of PIELD (Interlaminar Endoscopy) in the largest series of CES (n=9) also developed new transient motor weakness^10^. Moreover, for the treatment of upper LDH, PTELD has been more recommended^29, 30^. In our series, we had three cases of upper LDH, and they both showed complete recovery.

The recovery in CES may take months to years, as reported conventionally^3, 31^. A three-case report of anterior lumbar surgery and another report of endoscope assisted OLIF (Oblique lumbar Interbody Fusion) in CES, report a dramatic quick recovery in bladder function^32, 33^. Although these are relatively open surgery, this should raise a prudent question of an underestimated manipulative injury in posterior surgeries. The excellent outcome of bladder recovery in our series may be because the ventral access surgery could be executed at the earliest possible instance.

Post-surgical pain is the biggest concern before most surgeries. It invades and erodes wellbeing and increases anxiety-stress of the patient^17^. Moreover, loss of sexual function and significant physical disability also looms heavily on CES patients^1-4^. The recovery of bladder function happened within 48 hours in all but one patient in our study. The minimal pain after PTELD would be a positive factor towards a faster recovery. Comparatively, in conventional surgeries, only 50-70% of patients get either a true recovery or compensatory balanced outcome of bladder function^3^.

The medical comorbidities are not a contraindication for PTELD under LA^8, 11^. In fact, the negativities of GA on the patient’s visceral function (POUR-post-operative urinary retention) and cognitive dysfunction are not hidden and may have some critical role in immediate post-operative period in spine surgeries^34^. One patient of our series was operated under GA and he took one week to recover his bladder function. So, this aspect is completely avoided in CES patients operated under LA. Anaesthesiologist presence is mandatory to cover any emergency as well as to provide better communication.

However, there are negatives to PTELD and medico-legal implications of CES that need to be considered. Following PTELD post-operative, residual shadows may appear like pseudo-hernia. Lack of complete professional knowledge of competing surgeons and reporting by radiologists can cause disputes, especially if the patient has got residual or no recovery^10, 11^. Another negative aspect is radiation exposure^35, 36^.

Dural tears, exiting nerve injuries, and dysesthesia can also occur in PTELD^37^. Our results are equally better as compared with other PTELD short series studies on objective parameters and the speed of complete recovery (Table III). The statistically significant improved ODI, VAS and patient satisfaction index are remarkable. Though no NILBP was noted in any patient, there were nine patients with ILBP. This necessitates that mandatory lifestyle modification needs to be followed by all patients.

**Table III:** Comparative table of PTELD literature series in CES

Author Year	Number of patients	Bladder/ Bowel symptoms	CES classification	Bladder symptoms recovery After surgery	Bladder symptoms recovery Time	Follow-up (months)	Remarks
Kim (2007)^7^	1	Voiding difficulties	CESR	N/A	N/A	3	Patient was pregnant
Jha (201 5)^9^	1	Bladder dysfunction Saddle anaesthesia	N/A	Full recovery	Immediate	3	-
Li (2016)^10^	7 (Total n=16) There were 9 patients of PIELD.	Bladder/rectal dysfunction in 4 patients Saddle anaesthesia in 16 patients	Early and middle stages of Shi's Classification	Residual saddle anaesthesia in 3 patients	N/A	24-32	One patient developed motor weakness, and one patient developed an ipsilateral recurrent herniation.
Kim (2018)^8^	1	Saddle anaesthesia. Difficulty in urine passing with no urine incontinence	N/A	Full recovery	2 months	2	-
Namboothiri (2016)^11^	2	Saddle anaesthesia Bladder/rectal dysfunction	N/A	Full recovery	N/A	9-12 months	Biportal bilateral approach needed in both patients
Mahesha (2017)^12^	2	N/A	N/A	Full recovery	N/A	24	-
	(Total n=100)						
Gu (2017)^13^	2	Voiding dysfunction	N/A	Full recovery	1 day	24	
	(Total n=209)		-				
Present series	15	Classic Fraser *et al* criteria symptoms^4^	CESI(n=10) CESR(n=5)	Full Recovery	1.5 day	20.33	Largest series. Bilateral Biportal approach needed in two patients.

N/A: Details not available, CES: Cauda equina syndrome, PTELD / PIELD: percutaneous transforaminal/interlaminar endoscopic lumbar discectomy, CESI: incomplete cauda equina syndrome, CESR: complete cauda equina syndrome Retention, Total: Series patient numbers and not the CES patient numbers.

**Fig. 3:**
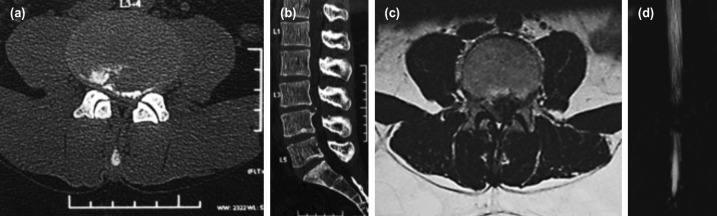
(a) Pre-operative CT (Computed Tomography) scan axial (b) and sagittal, (c) axial MRI in a 23-year-old male patient of CES with bilateral leg affection and vesicular dysfunction showing a calcified central LDH (Lumbar Disc Herniation). (d) MRI myelogram showing complete block.

**Fig. 4:**
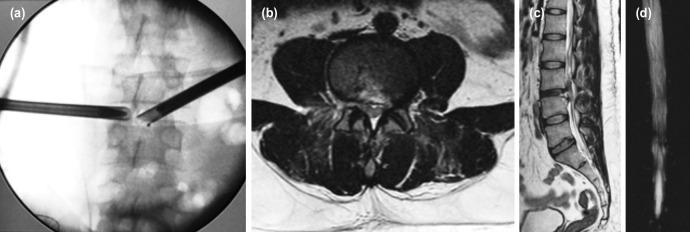
(a) “Inside out” Bi-lateral Bi-portal approach taken for surgery. (b,c) Post-operative T 2 axial and saggital MRI showing complete decompression. (d) MR myelogram shows blockage cleared and the patient recovered within one day.

In 13 out of 15 cases, basic techniques of surgery worked and BLBP approach was needed in two of our cases (Fig. 3, 4). This additional approach was also identified in one of the previous studies^11^. Rajasekaran *et al* suggest a conceptual change to understand that the disc prolapsed material can have associated endplate cartilage or/ and annulus in addition to nucleus fragment^38^. These fragments may be collagenized or calcified (CC) and must be removed to achieve complete decompression as was done in three of our cases.

There are many limitations to our study by its retrospective methodology itself. The study was a non-consecutive series. It is possible that less affected patients got enrolled in our present study even though our patients fitted into classic Fraser *et al* description of CES, and we followed Gleave *et al* classification of CESR and CESI^4, 23^. The patient feedback for bladder dysfunction was purely subjective, and no supportive urodynamic studies were conducted to document and compare. However, it is also well established that patients may be asymptomatic despite the severe disruption of bladder function in urodynamic studies^3, 39^. Moreover, in studies of surgery for CES, there exists a significant heterogeneity of reported outcomes^1-4, 22^. This indicates a clear need for the development of a Core Outcome Set (COS), which has been suggested with the involvement of patient inputs, for uniformity of database^22^.

## Conclusion

The superiority and curative outcome of the PTELD in CES is visible but more studies are needed before it is recommended for all cases due to the medico-legally volatile nature of the disabling condition.
